# 410. *Acinetobacter baumannii* Infections During COVID: Molecular Epidemiology and Outcomes at a Tertiary Hospital in Dallas

**DOI:** 10.1093/ofid/ofad500.480

**Published:** 2023-11-27

**Authors:** Afeefah F Khazi-Syed, Christine A Pybus, Andrew E Clark, Jiwoong Kim, Xiaowei Zhan, Dhara Desai, Jackson Barth, David E Greenberg

**Affiliations:** UT Southwestern Medical School, Arlington, Texas; UT SOUTHWESTERN MEDICAL CENTER, DALLAS, TX; University of Texas Southwestern Medical Center, Dallas, Texas; University of Texas Southwestern, Dallas, TX; UTSouthwestern Medical Center, Dallas, TX; University of Texas Southwestern Medical Center, Dallas, Texas; Southern Methodist University, Dallas, Texas; UT Southwestern, Dallas, Texas

## Abstract

**Background:**

*Acinetobacter baumannii* (AB) is a significant human pathogen that is frequently antibiotic resistant and has been associated with post-COVID-19 infections.

**Methods:**

In this study, we performed whole-genome sequencing on AB isolates collected at an academic medical center in Dallas, TX between January 2020 and June 2021. We extracted clinical and laboratory data and analyzed both outcomes and molecular epidemiology of AB isolates in patients with and without COVID-19.

**Results:**

Nineteen isolates collected from 17 AB-infected patients were included in this study. Most isolates were cultured from the respiratory tract (58%), followed by urine (16%), blood (10.5%), wound (10.5%), and catheter tips (5%). 4/17 patients were COVID-19 positive. The average Charlson Comorbidity Index at admission was lower for patients with COVID-19 (2.75) compared to counterparts (4.46). Using Fisher’s exact test, no statistical significance was observed in hospital events: ICU Admission (0.08), Ventilation (1.0), and Sepsis (0.23). The mortality rate during admission was 50% for COVID-19 patients and 23% for patients without COVID-19 (Odds Ratio: 3.33, p-value: 0.54, 95% CI: [0.32, 34.83]). All 19 isolates were carbapenem-resistant (CRAB) and multi-drug resistant (MDR) per definitions established by Magiarkos et al. 67% of isolates from COVID-19 patients were extensively-drug resistant (XDR) compared to 33% of isolates from non-COVID-19 patients. MIC data for cefiderocol was available for 9/19 isolates. Values ranged from 0.5 – 16 μM with 4/9 isolates being < 4 μM. Meropenem MIC values for all isolates were ≥ 64 μM. Many isolates contained at least one carbapenemase: OXA-72 (15/19), OXA-23 (3/19), OXA-95 (3/19), OXA-24 (1/19). MLST analysis revealed ST2 as the most common in all patients (15/19), followed by ST499 (3/19) and ST406 (1/19).

**Conclusion:**

COVID-19 patients infected with AB trended towards a significant difference in mortality compared to AB patients without documented COVID-19 during the same period. All AB isolates were CRAB, and isolates from COVID-19 patients were more likely to be XDR.

**Disclosures:**

**David E. Greenberg, MD**, University of Texas Southwestern Medical Center: Dr. Greenberg has numerous patents on PPMOs

